# Severe hearing impairment and risk of depression: A national cohort study

**DOI:** 10.1371/journal.pone.0179973

**Published:** 2017-06-22

**Authors:** So Young Kim, Hyung-Jong Kim, Eun-Kyu Park, Jiwon Joe, Songyong Sim, Hyo Geun Choi

**Affiliations:** 1 Department of Otorhinolaryngology-Head & Neck Surgery, CHA Bundang Medical Center, CHA University, Seongnam, Korea; 2 Department of Otorhinolaryngology-Head & Neck Surgery, Hallym University College of Medicine, Anyang, Korea; 3 Department of Statistics, Hallym University, Chuncheon, Korea; Universidad de Salamanca, SPAIN

## Abstract

**Objective:**

Hearing impairment is suggested to be associated with depression in the elderly. The present study evaluated the risk of depression after hearing impairment in all age groups matched by age, sex, income, and region of residence.

**Methods:**

The Korean Health Insurance Review and Assessment Service—National Patient Samples were collected for a period from 2002 to 2013. Hearing impairment was defined as a hearing threshold ≥ 60 dB in both ears or as ≥ 80 dB in one ear and ≥ 40 dB in one ear. Hearing-impaired participants performed a pure tone audiometry test 3 times and an auditory brainstem response threshold test once. The 6,136 hearing-impaired participants were matched 1:4 with 24,544 controls with no reported hearing impairment for age, sex, income, and region of residence. Depression was investigated based on the International Classification of Disease-10 codes F31 (bipolar affective disorder) through F39 (unspecified mood disorder) by a psychiatrist from 2002 through 2013. The crude (simple) and adjusted (age, sex, income, region of residence, dementia, hypertension, diabetes, and dyslipidemia) hazard ratio (HR) of hearing impairment on depression were analyzed using Cox-proportional hazard model.

**Results:**

The rate of depression was significantly higher in the severe hearing-impaired group than in the control group (7.9% vs. 5.7%, P < 0.001). Severe hearing impairment increased the risk of depression (adjusted HR = 1.37, 95% confidence interval [CI] = 1.24–1.52, P < 0.001). In a subgroup analysis, young (0–29 years old), middle-aged (30–59 years old), and old (≥ 60 years old) severe hearing-impaired groups showed significantly increased risk of depression compared to controls with no reported hearing impairment. In accordance with income level, severe hearing impairment elevated depression in the low and high income groups, but not in the middle income group.

**Conclusion:**

Severe hearing impairment increased the risk of depression independently of age, sex, region, past medical histories, and income (in low and high income persons but not in middle income persons).

## Introduction

Hearing impairment is one of the most common disorders, with prevalences of 22.73% and 21.7% in the general Korean and US populations, respectively [1,2]. In addition, 9.2% of Korean adults showed moderate-to-profound hearing impairment [3]. Although there have been some conflicts according to age and sex, the contribution of hearing impairment to depression has been reported [2,4,5]. Both audiometric hearing impairment and self-reported hearing impairment demonstrated significant relationships with depression in the adult population [2]. Although most studies considered older patients, young patients also showed an association between hearing impairment and depression in a few studies [6,7]. Hearing impairment was related to depressive symptoms independently of age, sex, degree of hearing loss, and socioeconomic levels [6]. Furthermore, it was reported that patients with sudden sensorineural hearing loss showed a 2.18-fold (95% confidence interval [CI] = 1.55–3.07) higher rate of depression than those in the normal hearing control group [7]. In particular, younger patients (< 65 years old) demonstrated a higher risk ratio of 2.64 (95% CI = 1.73–4.00) than older patients (≥ 65 years old) (risk ratio = 1.36, 95% CI = 0.62–2.75) [7]. Therefore, the effect of hearing impairment on depression should be considered in the young population as well as the older population.

The relationship between hearing impairment and depression is thought to be bidirectional. Hearing impairment has been suggested to be related to impaired social communication and cognitive function, thereby resulting in social isolation in the elderly [5,8,9]. These adverse effects of hearing impairment on social isolation and cognitive function could mediate a health-related impairment of quality of life, including increased anxiety or depression and adverse effects on activities of daily living [4]. Conversely, emotional factors, such as stress or depression, could induce hearing impairment [10,11]. Recently, depression was suggested to increase the risk of sudden sensorineural hearing loss with a 1.45 (95% CI = 1.12–1.86, P = 0.004) odds ratio [12]. Thus, the possible reciprocal effects between hearing impairment and depression should be considered to evaluate the impact of hearing impairment on depression.

Although previous studies reported the adverse effects of hearing impairment on depression, most of these studies were cross-sectional studies that could not delineate the causal relationship between hearing impairment and depression [2,4,5]. A few studies with a prospective study design were confined to the elderly population or based on self-reported hearing discomfort [13]. The present cohort study aimed to investigate the impact of hearing impairment on depression using a large, nation-wide, population based data. To discriminate the causal relationship between hearing impairment and depression, the depression which was diagnosed before the diagnosis of hearing impairment was excluded in this study design.

## Materials and methods

### Study population and data collection

The ethics committee of Hallym University (2014-I148) approved the use of these data. Written informed consent was exempted by the Institutional Review Board.

This national cohort study relied on data from the Korean Health Insurance Review and Assessment Service—National Patient Sample (HIRA-NPS). The Korean National Health Insurance Service (NHIS) selects samples directly from the entire population database to prevent non-sampling errors. Approximately 2% of the samples (one million) were selected from the entire Korean population (50 million). These selected data can be classified at 1,476 levels (age [18 categories], sex [2 categories], and income level [41 categories]) using randomized stratified systematic sampling methods via proportional allocation to represent the entire population. After data selection, the appropriateness of the sample was verified by previous study [14]. The details of the methods used to perform these procedures are provided by the National Health Insurance Sharing Service [15]. This cohort database included (i) personal information, (ii) health insurance claim codes (procedures and prescriptions), (iii) diagnostic codes using the International Classification of Disease-10 (ICD-10), (iv) death records from the Korean National Statistical Office (using the Korean Standard Classification of disease), (v) socio-economic data (residence and income), and (vi) medical examination data for each participant over a period ranging from 2002 to 2013.

Because all Korean citizens are recognized by a 13-digit resident registration number from birth to death, exact population statistics can be determined using this database. It is mandatory for all Koreans to enroll in the NHIS. All Korean hospitals and clinics use the 13-digit resident registration number to register individual patients in the medical insurance system. Therefore, the risk of overlapping medical records is minimal, even if a patient moves from one place to another. Moreover, all medical treatments in Korea can be tracked, without exception, using the HIRA system. In Korea, the notice of a death to an administrative entity is legally required before a funeral can be held.

### Participant selection

Out of 1,025,340 cases with 114,369,638 medical claim codes, we included participants who were registered as a hearing-impaired person in the Ministry of Health and Welfare. Hearing impairment was defined as having a hearing threshold ≥ 60 dB in both ears or ≥ 80 dB in one ear and ≥ 40 dB in the other. In Korea, to be registered as a hearing-impaired person, one must be checked three times by a pure tone audiometry test (PTA) and once by an auditory brainstem response. The average hearing threshold of PTA was calculated as follows: (500 Hz + 2*1000 Hz +2* 2000 Hz + 4000 Hz)/6.

The hearing-impaired participants were matched 1:4 with the participants among this cohort who were never diagnosed as having a hearing impairment or other disabilities (control group) from 2002 through 2013. The matches were processed by age, group, sex, income group, and region of residence. To prevent selection bias when selecting matched participants, the control group participants were sorted using a random number order and were then selected from top to bottom. It was assumed that the matched control participants were involved at the same time of the matched hearing-impaired participants. Therefore, the control group participant who died before the involvement time of the matched hearing-impaired participant was excluded. The participants who had a history of depression before hearing impairment were excluded in this study (n = 294). The hearing-impaired participants for whom we could not identify enough matching participants were excluded (n = 95).

**Fig 1:**
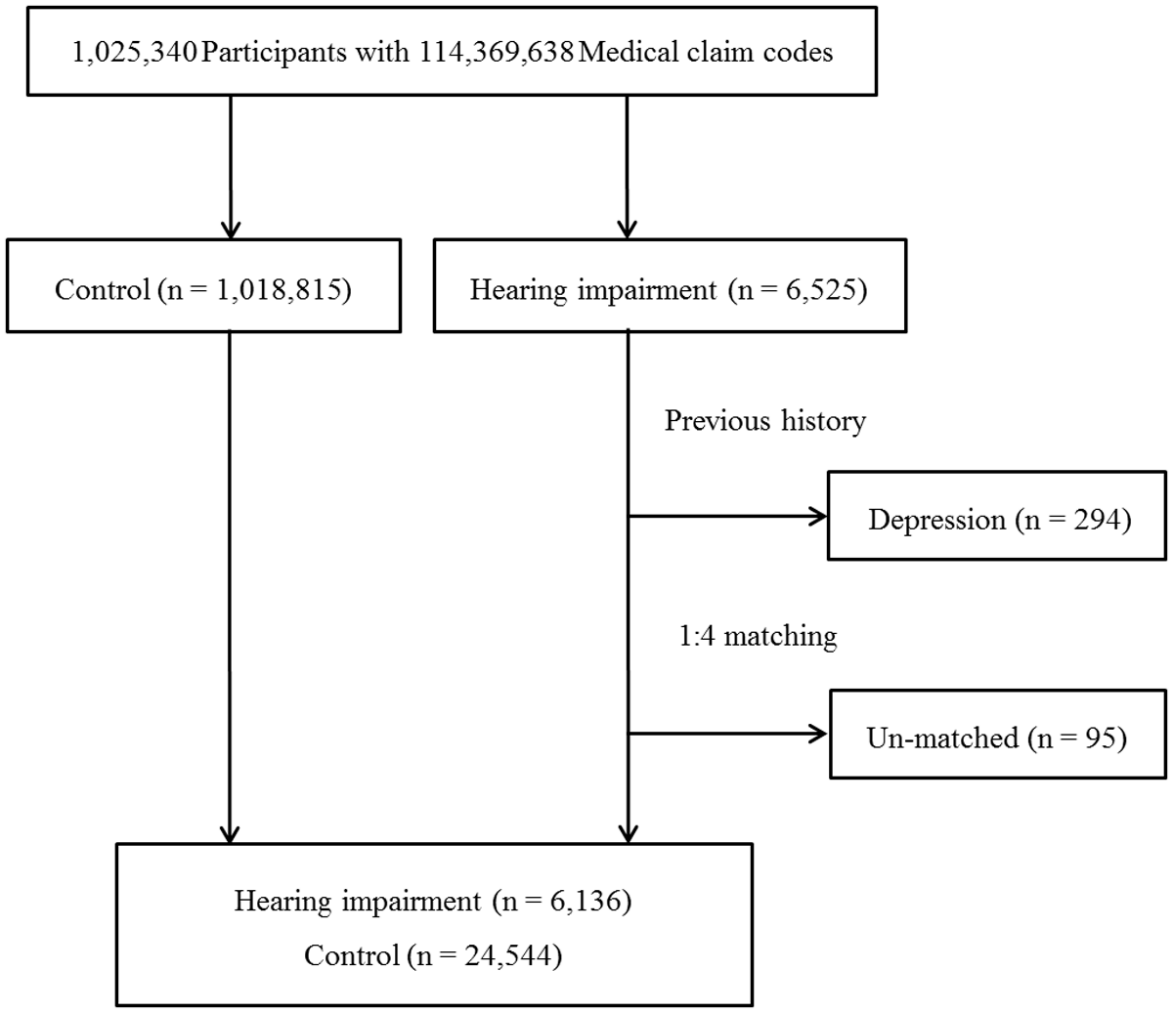
A schematic illustration of participant selection in the present study. Among a total of 6,525 hearing impaired participants, participants who had a history of depression before hearing impairment (n = 294) and could not find enough matching participants (n = 95) were excluded. The data for the 6,136 of hearing impaired participants and 24,544 control participants were analyzed.

Finally, 1:4 matching resulted in the inclusion of 6,136 of hearing impaired participants and 24,544 control participants (Fig 1). However, they were not matched for their past medical histories (dementia, hypertension, diabetes, and dyslipidemia).

### Variables

The age groups were classified using 5-year intervals: 0–4, 5–9, 10–15…, and 85+ years old. A total of 18 age groups were designated. The income groups were initially divided into 41 classes (one health aid class, 20 self-employment health insurance classes, and 20 employment health insurance classes). These groups were re-categorized into 11 classes (class 1 [lowest income]—11 [highest income]). The region of residence was divided into 16 areas according to the administrative district. These regions were regrouped into urban (Seoul, Busan, Daegu, Incheon, Gwangju, Daejeon, and Ulsan) and rural (Gyeonggi, Gangwon, Chungcheongbuk, Chungcheongnam, Jeollabuk, Jeollanam, Gyeongsangbuk, Gyeongsangnam, and Jeju) areas.

The past medical histories of participants were evaluated using ICD-10 codes. For the accuracy of diagnosis, dementia (F00 and G30), hypertension (I10 and I15), diabetes (E10-E49), and dyslipidemia (E78) were checked if the participants were treated ≥ 2 times.

Depression was defined using the ICD-10 codes F31 (bipolar affective disorder) through F39 (unspecified mood disorder) by a psychiatrist from 2002 through 2013.

### Statistical analyses

To analyze the hazard ratio of hearing impairment on depression, Cox-proportional hazard model was used. In this analysis, crude (simple) and adjusted (age, sex, income, region of residence, dementia, hypertension, diabetes, and dyslipidemia) model was used. 95% confidence interval (CI) were calculated.

For the subgroup analysis, we divided the participants with age and sex (0–29 year old, 30–59 year old, 60–85+ years old, male, and female). In another subgroup analysis, we grouped participants according to income level (low income [1–4], middle income [5–8], and high income [9–11]). Two-tailed analyses were conducted, and P values less than 0.05 were considered to indicate significance. The results were statistically analyzed using SPSS v. 21.0 (IBM, Armonk, NY, USA).

## Results

The distributions of age, sex, income level, and region of residence were comparably matched between the hearing-impaired and control groups (Table 1). The crude and adjusted hazard ratio (HR) were 1.40 (95% CI = 1.26–1.53) and 1.37 (95% CI = 1.23–1.52), respectively (P < 0.001) (Table 2). In total, 7.9% (487/5,649) of the hearing-impaired group was diagnosed with depression, which was significantly more than 5.7% (1,407/23,136) of the control group (P < 0.001) (S1 Table).

**Table 1:** General characteristics of participants.

Characteristics	Total participants	
	Hearing impairment (n, %)	Control group (n, %)
Age (years old)		
0–4	43 (0.7)	172 (0.7)
5–9	39 (0.6)	156 (0.6)
10–14	56 (0.9)	224 (0.9)
15–19	52 (0.8)	208 (0.8)
20–24	87 (1.4)	348 (1.4)
25–29	124 (2.0)	496 (2.0)
30–34	153 (2.5)	612 (2.5)
35–39	245 (4.0)	980 (4.0)
40–44	383 (6.2)	1,532 (6.2)
45–49	430 (7.0)	1,720 (7.0)
50–54	495 (8.1)	1,980 (8.1)
55–59	629 (10.3)	2,516 (10.3)
60–64	823 (13.4)	3,292 (13.4)
65–69	879 (14.3)	3,516 (14.3)
70–74	781 (12.7)	3,124 (12.7)
75–79	575 (9.4)	2,300 (9.4)
80–84	250 (4.1)	1,000 (4.1)
85+	92 (1.5)	368 (1.5)
Sex		
Male	3,428 (55.9)	13,712 (55.9)
Female	2,708 (44.1)	10,832 (44.1)
Income		
1 (lowest)	655 (10.7)	2,620 (10.7)
2	572 (9.3)	2,288 (9.3)
3	396 (6.5)	1,584 (6.5)
4	519 (8.5)	2,076 (8.5)
5	463 (7.5)	1,852 (7.5)
6	499 (8.1)	1,996 (8.1)
7	451 (7.4)	1,804 (7.4)
8	542 (8.8)	2,168 (8.8)
9	559 (9.1)	2,236 (9.1)
10	695 (11.3)	2,780 (11.3)
11 (highest)	785 (12.8)	3,140 (12.8)
Region of residence		
Urban	2,472 (40.3)	9,888 (40.3)
Rural	3,664 (59.7)	14,656 (59.7)
Dementia		
Yes	482 (7.9)	1,487 (6.1)
No	5,654 (92.1)	23,057 (93.9)
Diabetes mellitus		
Yes	1,501 (24.5)	6,025 (24.5)
No	4,635 (75.5)	18,519 (75.5)
Hypertension		
Yes	3,156 (51.4)	12,678 (51.7)
No	2,980 (48.6)	11,866 (48.3)
Dyslipidemia		
Yes	1,517 (24.7)	6,698 (27.3)
No	4,619 (75.3)	17,846 (72.7)

**Table 2:** Crude and adjusted hazard ratios (95% confidence interval) of hearing impairment for depression.

Characteristics	Depression			
	Crude	P-value	Adjusted†	P-value
Hearing impairment	1.40 (1.26–1.53)	< 0.001*	1.37 (1.23–1.52)	< 0.001*
Control	1.00		1.00	

* Cox-proportional hazard regression model, Significance at P < 0.05

^†^ Adjusted model for age, sex, income, region of residence, dementia, hypertension, diabetes, and dyslipidemia.

In a subgroup analysis, hearing impairment elevated the risk of depression in all of the subgroups except for the 30- to 59-year-old male and 60–85+ year-old female groups (Table 3). The adjusted HR was 2.78 for the 0–29 year-old male group (95% CI = 1.35–5.62, P = 0.005). The 60–85+ year-old male showed adjusted HR of 1.45 (95% CI = 1.20–75, P = 0.005). Compared to 5.5% (399/6,813) of the control group with depression, 8.5% (149/1,654) of the hearing-impaired group showed depression in 60- to 85+-year-old males (P < 0.001) (S2 Table). The rate of depression was highest in the hearing-impaired group in 30- to 59-year-old females (11.0%, 104/840), which was significantly higher than the 7.7% (290/3,486) in the control group (P = 0.001).

**Table 3:** Subgroup analysis of crude and adjusted hazard ratios (95% confidence interval) of hearing impairment for depression according to age and sex.

Characteristics	Depression			
	Crude	P-value	Adjusted†	P-value
**Age (0–29 years old), Male (n = 1,170)**				
Hearing impairment	2.80 (1.38–5.67)	0.004*	2.78 (1.35–5.62)	0.005*
Control	1.00		1.00	
**Age (0–29 years old), Female (n = 835)**				
Hearing impairment	1.93 (1.07–3.50)	0.030*	2.00 (1.10–3.61)	0.024*
Control	1.00		1.00	
**Age (30–59 years old), Male (n = 6,955)**				
Hearing impairment	1.23 (0.93–1.63)	0.138	1.25 (0.94–1.65)	0.122
Control	1.00		1.00	
**Age (30–59 years old), Female (n = 4,720)**				
Hearing impairment	1.46 (1.17–1.83)	0.001*	1.47 (1.17–1.84)	0.001*
Control	1.00		1.00	
**Age (60–85+ years old), Male (n = 9,015)**				
Hearing impairment	1.51 (1.25–1.82)	< 0.001*	1.45 (1.20–1.75)	< 0.001*
Control	1.00		1.00	
**Age (60–85+ years old), Female (n = 7,985)**				
Hearing impairment	1.25 (1.03–1.51)	0.021*	1.18 (0.98–1.43)	0.086
Control	1.00		1.00	

* Cox-proportional hazard regression model, Significance at P < 0.05

^†^ Adjusted model for age, sex, income, region of residence, dementia, hypertension, diabetes, and dyslipidemia.

The impact of hearing impairment on depression was different according to income level (Table 4). Hearing impairment significantly increased depression in the low (adjusted HR = 1.25, 95% CI = 1.03–1.52, P = 0.022) and high (adjusted HR = 1.64, 95% CI = 1.40–1.93,P < 0.001) income groups, while the hearing-impaired and control groups demonstrated comparable rates of depression in the middle income group (P = 0.085) (S3 Table).

**Table 4:** Subgroup analysis of crude and adjusted hazard ratios (95% confidence interval) of hearing impairment for depression according to income.

Characteristics	Depression			
	Crude	P-value	Adjusted†	P-value
**Low Income (1–4 group, n = 10,710)**				
Hearing impairment	1.27 (1.05–1.54)	0.014*	1.25 (1.03–1.52)	0.022*
Control	1.00		1.00	
**Middle Income (5–8 group, n = 9,755)**				
Hearing impairment	1.21 (1.00–1.46)	0.048*	1.18 (0.98–1.42)	0.085
Control	1.00		1.00	
**High Income (9–11 group, n = 10,195)**				
Hearing impairment	1.71 (1.46–2.01)	< 0.001*	1.64 (1.40–1.93)	< 0.001*
Control	1.00		1.00	

* Cox-proportional hazard regression model, Significance at P < 0.05

^†^ Adjusted model for age, sex, income, region of residence, dementia, hypertension, diabetes, and dyslipidemia.

## Discussion

Severe hearing impairment significantly increased the risk of depression in all age ranges in the study population when age, sex, income, and region were matched. In addition, severe hearing impairment elevated the risk of depression in the low and high income groups, but not in the middle income group.

Severe hearing impairment was a risk factor of depression in the present study. In accordance with the present result, previous cross-sectional studies also reported a positive association between hearing impairment and depression [2,4]. Anxiety or depression was 1.84-fold (95% CI = 1.25–2.70) higher in hearing-impaired adults with tinnitus compared to normal hearing adults when adjusted for possible confounders [4]. Another cross-sectional study in the elderly reported that the prevalence of depression was 2.4-fold (95% CI = 1.7–3.2) higher in the self-reported moderate hearing-impaired group than in the excellent hearing group [2]. Children and adolescent groups with hearing impairment also reported higher emotional difficulties [16]. The mental problems in hearing-impaired children even persisted after cochlear implantation [17]. However, the associations between hearing impairment and depression were controversial or confined to specific subgroups in previous studies [2,9]. A prior study reported the non-significant association between hearing impairment and depression in the elderly [9]. Not only the cross-sectional study design but also the criteria of hearing impairment with > 25 dB hearing thresholds might obscure the adverse effect of hearing impairment on depression in that study. Indeed, the mild-to-moderate hearing impairment was suggested to have little influence on depression [2]. A longitudinal study demonstrated significant associations of self-reported hearing impairment with loneliness only in elderly men, and no significant relationship was identified between hearing impairment and depression [13]. However, this prior study lacks objective hearing measures. In fact, self-reported hearing impairment, irrespective of the patients’ objective hearing ability, was associated with depression [18]. Thus, the present study investigated the diagnosis of depression after the diagnosis of hearing impairment, and performed pure tone audiometry in the population with hearing impairment.

In the subgroup analysis, the adverse effects of severe hearing impairment on depression were consistent in all age and sex groups, except for the middle-aged male group and old-aged female group. Severe hearing impairment especially increased the risk of depression in the older male group in this study. It was possible that the larger study population of the elderly, compared to the younger population, resulted in higher statistical power in the older group. Similar to the present result, a cross-sectional study demonstrated the higher prevalence of depression in hearing-impaired elderly men (AOR = 2.22, 95% CI = 1.07–4.61) [19]. However, previous studies suggested conflicting results on the relationship between hearing impairment and depression in accordance with age and sex. Contrary to our result, in a cross-sectional study of the elderly population, only the female group showed a significant association between measured hearing impairment and depression (OR = 3.9, 95% CI = 1.3–11.3) [2]. The higher prevalence of depression in women (14.7%, 95% CI = 12.7–16.9) than in men (9.0%, 95% CI = 7.5–10.8) might influence the significant association between hearing impairment and depression in women. In addition, the diagnosis of depression was based on the 9-item Patient Health Questionnaire rather than on diagnosis by a clinician. The Blue Mountain Study also reported the higher prevalence of depression in hearing-impaired women but not in men (Adjusted OR [AOR] = 1.95, 95% CI = 1.15–3.31) [20]. The conflict with the present result could be attributed to the difference in the hearing-impaired group, which was defined according to a hearing threshold of > 25 dB in that study.

In the present study, severe hearing impairment significantly elevated depression in the low and high income groups, but not in the middle income group. Because the high income group might be more privileged for leisure activity and social activities, which needs auditory function, the adverse effects of hearing impairment could be magnified in the high income group. On the other hands, hearing impairment was suggested to be related to low educational level, unemployment, and economic hardship [21]. These adverse socioeconomic effects of hearing impairment might induce depression.

The present study extended past studies on the influence of hearing impairment on depression with several strengths. Foremost, this study is one of the largest nationwide population studies encompassing entire age groups. The data were based on a database that covers the entire population and were verified by a statistician. Moreover, by matching age, sex, income, and region of residence, confounding effects of these general characteristics on hearing impairment and depression were excluded. Matching income and region was crucial because these factors decide the availability of medical care. Using the Korean NHIS, income level, which determines the allocation of medical welfare, was precisely surveyed. In addition, subgroup analyses were conducted to estimate specific associations of each confounder. Hearing impairment was based on the thresholds of pure tone audiometry. The participants who had a history of depression before hearing impairment were excluded in this study, and depression was investigated using the medical records within HIRA. Thus, recall bias could be prevented in the present study. HIRA data encompass all citizens of the nation, without exception. Therefore, there were no missing participants, and the confounding effect of pre-existing depression before hearing impairment could be minimized.

The diagnosis of hearing impairment or depression could be delayed in some populations. In addition, audiometric hearing evaluation was not conducted in control group. However, the likelihood of a delayed or missed diagnosis of hearing impairment might be small because the diagnosed hearing-impaired person has considerable benefits in Korea, including health insurance. Another weak point of the present study is that only the moderately severe (≥ 60 dB) and more severe degrees of hearing impairment were counted in the present study. The severity of depression also was not discriminately considered in this study. Because whole age groups were included in this study, depression in young participants was also mixed in the present results. However, there were only 7 subjects with depression less than 10 years old, which might have no significant impact on the present results. Lastly, hearing rehabilitation using a hearing aid or cochlear implant could compensate for the effects of hearing impairment [22,23]. However, a prior study suggested that the relationship of hearing impairment with depression was independent of the improved cognitive function that resulted from hearing aid use [5]. In addition, the proportion of individuals wearing hearing aids or cochlear implants is as low as approximately 12.6% in Korea [24].

## Conclusion

Severe hearing impairment increased the risk of depression in the overall population in Korea. Severe hearing impairment was a risk factor for depression in the young, as well as in the older population. The significant effect of severe hearing impairment on the rate of depression was noted in the low and high income groups, but not in the middle income group.

## Supporting information

S1 Table The rate of depression between hearing loss and control group during follow up. (DOCX)

S2 Table Subgroup analysis of the rate of depression between hearing loss and control group during follow up according to age and sex. (DOCX)

S3 Table Subgroup analysis of the rate of depression between hearing loss and control group during follow up according to income. (DOCX)
