# Supplementary material for: Severe hearing impairment and risk of depression: A national cohort study
Source: PLoS One. 2017 Jun 22;12(6):e0179973. doi: 10.1371/journal.pone.0179973 (PMC5481021; doi:10.1371/journal.pone.0179973)
Supplement: S1 Table — (DOCX) [file pone.0179973.s001.docx]

**S1 Table** The rate of depression between hearing loss and control group during follow up

| Depression | Hearing impairment | Control group | P-value |
| --- | --- | --- | --- |
| Normal (n, %) | 5,649 (92.1%) | 23,136 (94.3%) | < 0.001* |
| Depression (n, %) | 487 (7.9%) | 1,407 (5.7%) |  |

* Chi-square test. Significance at P < 0.05
