# Supplementary material for: Severe hearing impairment and risk of depression: A national cohort study
Source: PLoS One. 2017 Jun 22;12(6):e0179973. doi: 10.1371/journal.pone.0179973 (PMC5481021; doi:10.1371/journal.pone.0179973)
Supplement: S2 Table — (DOCX) [file pone.0179973.s002.docx]

**S2 Table** Subgroup analysis of the rate of depression between hearing loss and control group during follow up according to age and sex

|  | Hearing impairment | Control group | P-value |
| --- | --- | --- | --- |
| **Age (0-29 years old), Male** |  |  |  |
| Normal (n, %) | 221 (94.4%) | 917 (98.0%) | 0.003* |
| Depression (n, %) | 13 (5.6%) | 19 (2.0%) |  |
| **Age (0-29 years old), Female** |  |  |  |
| Normal (n, %) | 151 (90.4%) | 634 (94.9%) | 0.029* |
| Depression (n, %) | 16 (9.6%) | 34 (5.1%) |  |
| **Age (30-59 years old), Male** |  |  |  |
| Normal (n, %) | 1,326 (95.3%) | 5,352 (96.2%) | 0.141 |
| Depression (n, %) | 289 (4.7%) | 212 (3.8%) |  |
| **Age (30-59 years old), Female** |  |  |  |
| Normal (n, %) | 840 (89.0%) | 3,486 (92.3%) | 0.001* |
| Depression (n, %) | 104 (11.0%) | 290 (7.7%) |  |
| **Age (60-85+ years old), Male** |  |  |  |
| Normal (n, %) | 1,654 (91.7%) | 6,813 (94.5%) | < 0.001* |
| Depression (n, %) | 149 (8.3%) | 399 (5.5%) |  |
| **Age (60-85+ years old), Female** |  |  |  |
| Normal (n, %) | 1,457 (91.2%) | 5,934 (92.9%) | 0.023* |
| Depression (n, %) | 140 (8.8%) | 453 (7.1%) |  |

* Chi-square test. Significance at P < 0.05
