# Supplementary material for: Severe hearing impairment and risk of depression: A national cohort study
Source: PLoS One. 2017 Jun 22;12(6):e0179973. doi: 10.1371/journal.pone.0179973 (PMC5481021; doi:10.1371/journal.pone.0179973)
Supplement: S3 Table — (DOCX) [file pone.0179973.s003.docx]

**S3 Table** Subgroup analysis of the rate of depression between hearing loss and control group during follow up according to income

|  | Hearing impairment | Control group | P-value |
| --- | --- | --- | --- |
| **Low Income (1-4 group)** |  |  |  |
| Normal (n, %) | 2,005 (93.6%) | 8,135 (94.9%) | 0.013* |
| Depression (n, %) | 137 (6.4%) | 433 (5.1%) |  |
| **Middle Income (5-8 group)** |  |  |  |
| Normal (n, %) | 1,812 (92.7%) | 7,343 (93.9%) | 0.049* |
| Depression (n, %) | 143 (7.3%) | 477 (6.1%) |  |
| **High Income (9-11 group)** |  |  |  |
| Normal (n, %) | 1,832 (89.8%) | 7,658 (93.9%) | <0.001* |
| Depression (n, %) | 207 (10.2%) | 497 (6.1%) |  |

* Chi-square test. Significance at P < 0.05
